# Medical News and Bulletin

**Published:** 1843-03-04

**Authors:** 


☞ We have been compelled to omit the Clinics of the University of Pennsylvania, and the Jefferson Medical College, on account of our foreign selections for this number.

FOREIGN PHYSICIANS IN PARIS.

The number of foreign physicians now authorised to practice in Paris amounts to thirty-one. Within the last ten years only two or three permissions have been granted, in spite of the numerous demands addressed to the Minister of Public Instruction.

DEATH OF MR. WALKER.

This distinguished Surgeon died in London, on the 2d of January, after a severe illness of four days. Mr. W. was, at the time of his death, one of the Surgeons of St. George's Hospital.

DR. LOUIS.

This celebrated observer has been promoted to the rank of officer of the Legion of Honour.

PROTESTANT SISTERS OF CHARITY.

A society was formed in London, in 1840, whose object is to provide all classes with nurses of a superior stamp to those commonly to be met with. The donations of rich patients are to support the sisters, both for the benefit of the rich and the poor. Before a candidate can be enrolled on the list of sisters, she undergoes a probation of two months in a public hospital, where her knowledge is increased and her powers of endurance tested. Much as knowledge is desirable in a nurse, good feeling ranks much higher; and the object of the society is to procure sisters who will devote themselves to their duties from charity, in the highest sense of the word, rather than for the hope of gain.

"The sisters receive a stipend, and during the intervals of their engagements reside at home, (in Devonshire Street, Bishopsgate,) where they employ a part of their time in nursing the sick poor of the neighbourhood.

"They are dressed in a uniform, and 'in no case wear gold ornaments or jewellery, lace, embroidery, feathers, or artificial flowers.' Their engagement is entered into for three, five, or seven years; but if a sister wishes, for some good reason, to resign her office sooner, she is to give three months' notice. The pamphlet contains the names of twelve sisters; but as the list in the copy before us has two erasures, and five manuscript additions, we may presume the present number to be fifteen. The sisters are forbidden to accept any remuneration; the sums paid being sent to the board of management.

"They are also charged most earnestly to hold sacred the knowledge which, to a certain extent, they must obtain of the private affairs of any household or individual they may attend."

AN EASY METHOD OF OBTAINING A LITERARY REPUTATION.

Several years since a Dr. Jonathan Green published in England, as an *original* work, a "Compendium of the Diseases of the Skin," and dedicated it to Sir Henry Halford. This work was highly lauded from one end of the kingdom to the other; republished in this country in one of the Library publications, and puffed generally over the Union. The British and Foreign Review told its readers that "Dr. Green's work was complete, and worthy of its author's good reputation." The able editor of the Medico-Chirurgical, Dr. James Johnston, affirmed that "Dr. Green's Observations on Impetigo and Porrigo were certainly the most judicious that we have ever read; they are derived from sound pathological views." Dr. Willis, the translator of Rayer, pronounced it "as a manual every way superior to Bateman, and as having the advantage of the *Abrégé Pratique* in having been written by an individual intimately acquainted with the subject." The Lancet, Medical Gazette, &c. were all loud in its praise. The recent translation of the "Abrégé Pratique des Maladies de la Peau" of MM. Cazenave and Schedel by Dr. Burgess, has opened the eyes of these sagacious and learned critics to one of the most extraordinary frauds ever perpetrated. This original work of Dr. Green is *a literal translation, from the beginning to the end*, of the work of MM. Cazenave and Schedel.

————————"foul deeds will rise,
Though all the earth overwhelm them to men's eyes."

DEATH OF DR. JOSEPH FRANK, OF VIENNA.

We have to announce with regret, the death, at Vienna, of Dr. Joseph Frank, son of the illustrious John Peter Frank, and author of the best treatise on the practice of medicine.

DEATH OF MR. KEMP, OF EDINBURGH.

This eminent man, the Lecturer on Practical Chemistry in the University of Edinburgh, died on the 28th of last November, at the early age of thirty-seven. His disease was aneurism of the arch of the aorta, which, for two weeks previous to his death, induced severe attacks of angina pectoris, and a sense of impending death. Mr. Kemp commenced his public lectures in 1829, but was previously known as a private lecturer on Chemistry. He was an early and ardent cultivator of science. To him we are indebted for the advantages of the amalgamizing of the zinc plates in the galvanic apparatus, an improvement now very generally adopted in the construction of the galvanic batteries. He experimented largely on the gases and on combustion.

HONORARY MEMBERS OF THE ROYAL ACADEMY OF MEDICINE, PARIS.

The Moniteur contains a list of the honorary members of the Royal Academy of Medicine, approved by the King. The following are the English physicians and surgeons named in the list:—Drs. Abercrombie, Bright, Sir James Clark, Samuel Cooper, W. Guthrie, Marshall Hall, B. Travers, and W. Lawrence.

